# Comparison of a New Multiplex Immunoassay for Measurement of Ferritin, Soluble Transferrin Receptor, Retinol-Binding Protein, C-Reactive Protein and α^1^-Acid-glycoprotein Concentrations against a Widely-Used s-ELISA Method

**DOI:** 10.3390/diagnostics8010013

**Published:** 2018-02-02

**Authors:** Crystal D. Karakochuk, Amanda M. Henderson, Kaitlyn L. I. Samson, Abeer M. Aljaadi, Angela M. Devlin, Elodie Becquey, James P. Wirth, Fabian Rohner

**Affiliations:** 1 Food, Nutrition, and Health, University of British Columbia, Vancouver, BC V6T 1Z4, Canada; kaitlyn.samson@ubc.ca (K.L.I.S.); aaljaadi@bcchr.ca (A.M.A.); 2 BC Children’s Hospital Research Institute, Vancouver, BC V5Z 4H4, Canada; ahenderson@bcchr.ca (A.M.H.); adevlin@bcchr.ca (A.M.D.); 3 Department of Paediatrics, University of British Columbia, Vancouver, BC V6H 3V4, Canada; 4 International Food Policy Research Institute, Washington, DC 20005, USA; e.becquey@cgiar.org; 5 GroundWork, Fläsch 7306, Switzerland; james@groundworkhealth.org (J.P.W.); fabian@groundworkhealth.org (F.R.)

**Keywords:** α^1^-acid glycoprotein, C-reactive protein, ELISA, ferritin, immunoassay, inflammation, micronutrient, retinol-binding protein, soluble transferrin receptor

## Abstract

Recently, a multiplex ELISA (Quansys Biosciences) was developed that measures ferritin, soluble transferrin receptor (sTfR), retinol-binding protein (RBP), C-reactive protein (CRP), α^1^-acid glycoprotein (AGP), thyroglobulin, and histidine-rich protein 2. Our primary aim was to conduct a method-comparison study to compare five biomarkers (ferritin, sTfR, RBP, CRP, and AGP) measured with the Quansys assay and a widely-used s-ELISA (VitMin Lab, Willstaett, Germany) with use of serum samples from 180 women and children from Burkina Faso, Cambodia, and Malaysia. Bias and concordance were used to describe the agreement in values measured by the two methods. We observed poor overall agreement between the methods, both with regard to biomarker concentrations and deficiency prevalence estimates. Several measurements were outside of the limit of detection with use of the Quansys ELISA (total *n* = 42 for ferritin, *n* = 2 for sTfR, *n* = 0 for AGP, *n* = 5 for CRP, *n* = 22 for RBP), limiting our ability to interpret assay findings. Although the Quansys ELISA has great potential to simplify laboratory analysis of key nutritional and inflammation biomarkers, there are some weaknesses in the procedures. Overall, we found poor comparability of results between methods. Besides addressing procedural issues, additional validation of the Quansys against a gold standard method is warranted for future research.

## 1. Introduction

Micronutrient deficiencies are thought to be widespread globally but particularly affect certain subgroups in low- and middle-income countries, such as young children and women of child-bearing age [1]. The measurement of nutritional biomarkers such as iron, vitamin A, iodine, folate, vitamin B_12_, and zinc, is an essential component of assessing nutritional status at the population-level. Such biomarker analyses are done in a laboratory, either in the country where samples were collected or internationally. In either case, acceptable precision and accuracy of laboratory analysis is of primary importance in obtaining reliable estimates of nutritional status. In addition, affordability is an important criterion for selecting laboratory tests to be done in a survey or study, in particular in resource-constrained settings. Other factors include sample throughput, complexity of the method, technical expertise required for operation and maintenance, and quantity of blood sample available for analysis. 

Often in large micronutrient surveys conducted in low- and middle-income countries, the identification of a suitable laboratory to measure selected biomarkers with adequate quality and affordable cost is not straightforward. When biological samples cannot be exported, stakeholders must identify a laboratory with sufficient expertise in-country, and it can be challenging to find a laboratory that has the needed capacity at affordable costs. When exporting samples is possible, affordability is also often a challenge.

In the recent past years, the VitMin Lab in Germany (led by Dr. Juergen Erhardt) has automated and scaled up an in-house sandwich ELISA (s-ELISA) [2], enabling the analysis of large numbers of samples at a very competitive cost. This laboratory offers the analysis of ferritin, soluble transferrin receptor (sTfR), retinol-binding protein (RBP), C-reactive protein (CRP), and α^1^-acid-glycoprotein (AGP). It has demonstrated high performance in external quality control schemes [2]. Because this laboratory offers outstanding quality at a low price, many groups working in the field of nutrition rely heavily on it for analysis of said biomarkers. These biomarkers are ideal for assessing iron and vitamin A status (ferritin, sTfR, RBP) and adjusting for their response to inflammatory triggers [3]. This strong performance and a low price has: (a) resulted in a high demand for the services of the single person operating the VitMin Lab; and (b) forced the laboratory to restrict the number of samples accepted per project to a somewhat arbitrary number of 3000 samples in order to cope with the huge demand. Repeated attempts of the laboratory to establish the methodology in other laboratories for analysing large number of samples have not yet been successful [4]. In addition, to our knowledge, the range of aforementioned biomarkers cannot currently be measured on a single commercially-available clinical analyser, further complicating the issue. Several models are available that measure all but either AGP or RBP, necessitating employment of several methods/analysers. Thus, while the VitMin Lab is a viable and reliable solution at present, it has the drawbacks of: (a) dependency on a single laboratory; (b) limitations in the number of samples that can be analysed; and (c) the permission and resources to export samples from the country of origin to Germany. Therefore, there is a need to identify alternative options. 

PATH, in collaboration with Quansys (Quansys Biosciences, Logan, UT, USA), has developed a multiplex ELISA (Q-Plex™ Human Micronutrient Assay) that measures the same five biomarkers as those measured by the VitMin Lab, as well as thyroglobulin and histidine-rich protein 2 (HRP2; a biomarker to detect recent or current malaria parasitemia). The multiplex platform is relatively easy to use and requires less sophisticated laboratory knowledge and skills than other methods, which would make it suitable for use in different contexts, including those in low- and middle-income countries. Additionally, although the multiplex reader is relatively expensive, the cost per sample of the disposable microplates is similar to that of the VitMin Lab. 

As a primary goal, we conducted a method-comparison study to compare five biomarkers (ferritin, sTfR, RBP, CRP, and AGP) measured by the VitMin Lab and using the Quansys assay with use of serum blood sampled from women and children from three different regions. As a secondary goal, we compared a sub-sample of children with confirmed qualitative malaria testing to the Quansys assay to determine its diagnostic ability to identify children with malaria.

## 2. Materials and Methods 

### 2.1. Description of the ELISA Methods 

The VitMin lab uses a s-ELISA that concurrently measures ferritin, sTfR, RBP, AGP, and CRP in a 384-well plate with capture antibody. Capture antibodies used were: ferritin (Code A0133, Dako), sTfR (Cat. No 4Tr26; Clone 23D10, Hytest), RBP (Code A0040, Dako), CRP (Code A0073, Dako, Denmark). Detection antibodies were: anti-ferritin-horseradish peroxidase (Code P0145, Dako), anti-sTfR- horseradish peroxidase (Cat. No. 4Tr26-c; Clone 13E4, Hytest), anti-RBP-anti-ferritin-horseradish peroxidase (Code P0304, Dako) and anti-CRP-anti-ferritin-horseradish peroxidase [2]. Serum controls (Liquicheck, Bio-Rad) were used as standards for the calibration curves [2]. Quality control (QC) samples were prepared from serum samples with a low and high content of analytes [2]. Full details on the s-ELISA assay can be found elsewhere [2]. 

The Quansys Q-Plex ELISA is a quantitative chemiluminescent assay that concurrently measures the same five analytes as Erhardt’s s-ELISA, as well as thyroglobulin and HRP2, in a 96-well plate. Each well concurrently measures the seven analytes along with a positive control. Binding of detection moieties is measured via the chemiluminescence produced by streptavidin horseradish peroxidase in the presence of a luminol-based substrate [5]. Capture and detector mouse anti-human IgA antibodies were used for ferritin (F23, F31, and 4F23, Hytest, Turku, Finland). Capture antibodies used for the other analytes included: sTfR (4Tr26 13E4, Hytest), RBP (RBP, 4RBP2, RB42, Hytest), CRP (4C28 C6, Hytest), and AGP (GW22927F, Sigma Aldrich) [5]. Purified ferritin (RP-87068, ThermoFisher, Waltham, MA, USA), sTfR (8Tr56, Hytest), CRP (8C72, Hytest), AGP (G9885, Sigma Aldrich, St. Louis, MO, USA), and RBP (8RF9, Hytest) antigens were used [5]. Serum controls (Liquicheck, Bio-Rad, Hercules, CA, USA) were used as standards for the calibration curves [5]. Full details on the Quansys Q-Plex ELISA assay can be found elsewhere [5].

### 2.2. Description of the Gold Standard Malaria Test

Qualitative malaria diagnosis was conducted by detecting the histidine-rich protein 2 (HRP2) antigen of *Plasmodium falciparum* using a commercial kit (SD BIOLINE Malaria Ag P.f, Standard Diagnostics, Gyeonggi-do, Republic of Korea) and was considered as the gold standard method (in the method comparison to the Quansys s-ELISA method) for detecting malarial infection that occurred in the last month [6]. 

### 2.3. Studied Population 

A summary of the origin of the 180 serum samples for the primary method comparison analysis is described in Table 1. The 60 samples from Burkina Faso children were included in the malaria indicator method-comparison in a separate analysis. Ethics approval was obtained from the International Food Policy Research Institute’s IRB (2014-7-PHND-M) and the National Ethics Committee of Burkina Faso (2014-02-015)for use of the Burkina Faso samples; from the University of British Columbia Clinical Research Ethics Board in Canada (H12-00451) and the National Ethics Committee for Health Research in Cambodia (010-NECHR) for use of the Cambodia samples; and from the University of British Columbia Clinical Research Ethics Board in Canada (H15-00521) and the Ethics Committee for Research involving Human Subjects at the University Putra Malaysia in Malaysia (UPM/TNCPI/RMC/1.4.18.1 [JKEUPM]/F2) for use of the Malaysian samples. 

### 2.4. Blood Collection Procedures

In Burkina Faso, a capillary blood sample was collected from the finger into silica-coated blood collection tubes (Microvette 300, Sarstedt, Nümbrecht, Germany) and stored and transported cold until later centrifugation on the same day at the district health centre in Fada-N’Gourma (East Region of Burkina Faso). Samples were stored frozen (−20 °C) until shipment to the VitMin lab (July 2016) and use on the Quansys platform (June 2017).

In Cambodia, a 3-h fasting venous blood sample was collected from women in the morning into a 3.5 mL trace element-free tube (Becton Dickinson, Franklin Lakes, NJ, USA) and transported on ice until time of processing (within ~4–6 h) at the National Institute of Public Health Laboratory in Phnom Penh, Cambodia. Samples were stored at −70 °C until shipment to Canada. In Canada, samples were stored at −80 °C until time of shipment to Erhardt’s VitMin lab for the s-ELISA (June 2015) and until time of Quansys ELISA (June 2017).

In Malaysia, a 10-h overnight fasting venous blood sample was collected from women in the morning into a 3.5 mL trace element-free tube (Becton Dickinson, Franklin Lakes, NJ, USA) and transported on ice until time of processing (within ~3–4 h) at the Faculty of Medicine and Health Sciences, University of Putra, Malaysia. Samples were stored at −20 °C until shipment to Canada via World Courier (Stamford, CT, USA). In Canada, samples were stored at −80 °C until time of shipment to Erhardt’s VitMin lab for the s-ELISA (October 2016) and until time of Quansys ELISA (June 2017).

### 2.5. Data and Statistical Analysis

Biomarkers concentrations were reported as mean ± SD or median (IQR) depending on the distribution (normal or skewed, respectively). Commonly-used cut-offs were used to define nutritional deficiencies were as follows: ferritin <12 μg/L for children and <15 μg/L for women [7], sTfR <8.3 mg/L [8], and RBP <0.7 μmol/L [9]. Acute inflammation was defined as CRP >5 mg/L and chronic inflammation as AGP >1 mg/L [10]. HRP2 >0.92 μg/L was used to indicate a positive malaria diagnosis in the Quansys ELISA (personal communication, A. Tyler, Quansys). All values were unadjusted (e.g., no adjustments for inflammation or other factors). 

Bland and Altman’s bias and limits of agreement (95% CI) were used to describe the agreement in values measured by the two methods [11,12]. Bias was defined as the difference in means between the two measures. Limit of agreement plots were also generated for visual interpretation. Lin’s concordance coefficient was also calculated as a measure of reproducibility between the two methods [13]. Concordance measures the departure of the measured values from a 45° line of perfect concordance. We also estimated linear trend equations in the form (*y* = a*x* + b) for each population group for the comparison between each of the two methods. Two-sided *p*-values < 0.05 indicated statistical significance. Stata software version SE/13.1 for Mac (Stata Corp., College Station, TX, USA) was used for analyses.

## 3. Results

### 3.1. Quality Control

Quality control data for the s-ELISA are described as overall co-efficients of variation (CV, %) of the different analytes for each population group (with expected concentrations). Calibration curves were adjusted with a control sample, measured in 10 wells with Biorad Liquichek controls in 3 different concentrations in 6 wells on each plate. CV’s for samples from the three countries were <3.2% for Ferritin, <4.3% for sTfR, <4.0% for RBP, <6.2% for CRP and <10.1% for AGP. The Quansys software provides the following quality control data in each measured plate (Table 2). 

The total number of samples that were outside of the LOD range in the Quansys ELISA were: *n* = 35/179 (20%) for ferritin, *n* = 2/180 (<1%) for sTfR, *n* = 0/180 (0%) for AGP, *n* = 7/180 (4%) for CRP, and *n* = 4/180 (2%) for RBP. One sample failed the s-ELISA analysis for ferritin in the Cambodian population; thus, only *n* = 59 samples were available for comparison in this group.

### 3.2. Characterisitcs of the Women and Children Included in the Analysis

A total of 180 individuals were included in the analysis from three countries: Burkina Faso, Cambodia, and Malaysia. Table 3 presents the mean or median concentrations of each nutritional biomarker and the deficiency prevalence rates of women and children included in the method-comparison analyses. 

Ferritin: Prevalence of iron deficiency based on low ferritin concentrations (<12 and <15 μg/L for children and women, respectively) varied between the s-ELISA and Quansys ELISAs in Burkina Faso children (17–32%), Cambodian women (0–23%), and Malaysian women (6–18%). Prevalence also varied between the Quansys ELISAs in Burkina Faso and Malaysia regardless of whether or not measurements outside of the limit of detection (LOD) were excluded or included (17–22% and 6–10%, respectively).

STfR: Prevalence of iron deficiency based on sTfR (>8.3 mg/L) varied between the two methods in Burkina Faso children (75–100%), Cambodian women (35–50%), and Malaysian women (8–17%). Prevalence also varied between the Quansys ELISAs in Malaysia regardless whether or not measurements outside of the LOD were excluded or included (10–17%).

AGP: Prevalence of chronic inflammation (>1 mg/L) varied between the two methods in Cambodian women (10–15%) and Malaysian women (3–25%), but was relatively similar in Burkina Faso children (65–67%).

CRP: Prevalence of acute inflammation (>5 mg/L) varied between the two methods in Burkina Faso children (42–58%) and Malaysian women (10–34%), but was relatively similar in Cambodian women (7–8%).

RBP: Prevalence of vitamin A deficiency based on RBP concentrations (<0.7 μmol/L) varied between the two methods in Burkina Faso children. No differences in vitamin A deficiency were observed in Cambodian and Malaysian women in either the s-ELISA or Quansys ELISA (2% and 0% prevalence, respectively).

### 3.3. Trend Estimates for Each Analyte for Each Population Group and the Pooled Population

We estimated linear trend equations in the form (*y* = a*x* + b; y representing the Quansys method) for each population group for the comparison between each of the two methods (Table 4). Equations were calculated for each analyte using all samples within the LOD range in the Quansys ELISA, with the exception of ferritin, for which we calculated two trend equations for (1) samples within the LOD range; and (2) all samples within and outside of the LOD range.

### 3.4. Method-Comparisons between the Two Methods for Each Analyte

Bias, limits of agreement, and correlation coefficients of ferritin, sTfR, AGP, CRP, and RBP concentrations comparing the s-ELISA and the Quansys ELISA kit are described in Table 5. Results are shown for all samples, as well as only for those within the LOD range in the Quansys ELISA. 

Ferritin: Agreement between the two methods was poor in the Burkina Faso, Cambodian and Malaysian populations (bias ranged from 14.7 to 29.3 μg/L, and concordance ranged from 0.40 to 0.62). Bias did not improve when the samples outside of the LOD range were excluded.

STfR: Agreement between the two methods was poor in the Burkina Faso children, (bias of 26.5 mg/L). Conversely, agreement was low in the Cambodian and Malaysian women (bias of 5.1 and 0.1 mg/L), respectively. Concordance ranged from 0.09 to 0.62 in the Burkina Faso and Cambodian women, but was higher (0.78) in the Malaysian women. Bias did not improve when samples outside of the LOD range were excluded.

AGP: Agreement between the two methods varied among the three populations (0.1–0.2 g/L in all three groups). Concordance ranged from 0.41 to 0.85. No samples were outside of the LOD range on the Quansys assay.

CRP: Agreement between the two methods varied among the three populations (1.4–3.1 mg/L in all three groups). Concordance was relatively good in all three populations and ranged from 0.74 to 0.79. Bias did not change when the samples outside of the LOD range were excluded.

RBP: Agreement between the two methods varied from −0.04 to 0.6 in the three populations. Concordance ranged from 0.38 to 0.83. Bias did not improve when the samples outside of the LOD range were excluded (although only 2 samples were excluded for this reason).

**Figure 1:**
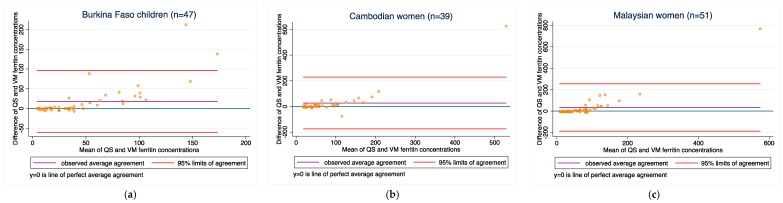
Ferritin concentrations for (**a**) Burkina Faso children (*n* = 47); (**b**) Cambodian women (*n* = 39); and (**c**) Malaysian women (*n* = 51). QS: ferritin results obtained on the Quansys ELISA; VM: ferritin results obtained from the VitMin lab.

**Figure 2:**
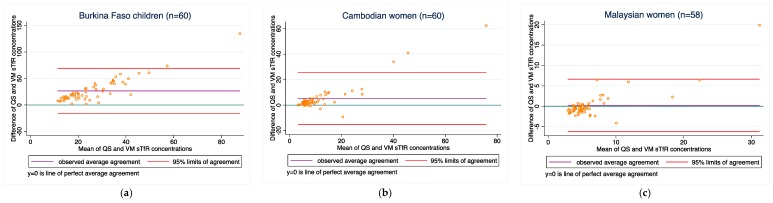
STfR concentrations for (**a**) Burkina Faso children (*n* = 60); (**b**) Cambodian women (*n* = 60); and (**c**) Malaysian women (*n* = 58).

**Figure 3:**
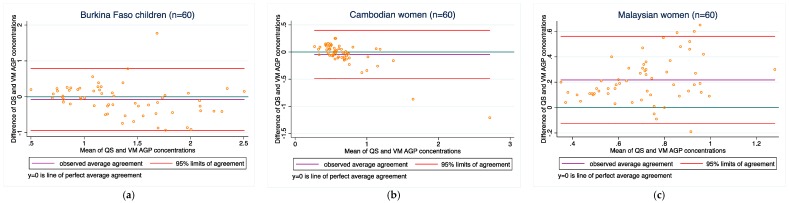
AGP concentrations for (**a**) Burkina Faso children (*n* = 60); (**b**) Cambodian women (*n* = 60); and (**c**) Malaysian women (*n* = 60).

**Figure 4:**
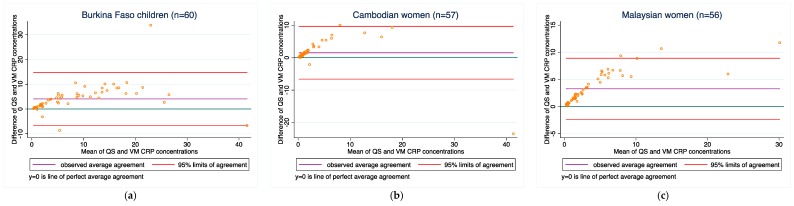
CRP concentrations for (**a**) Burkina Faso children (*n* = 60); (**b**) Cambodian women (*n* = 57); and (**c**) Malaysian women (*n* = 56).

**Figure 5:**
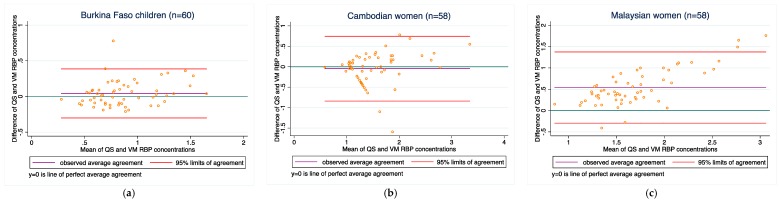
RBP concentrations for (**a**) Burkina Faso children (*n* = 60); (**b**) Cambodian women (*n* = 58); and (**c**) Malaysian women (*n* = 58).

Figure 1, Figure 2, Figure 3, Figure 4 and Figure 5 present the bias and limit of agreement plots for each of the five analytes in each population group for samples within the LOD range (excludes all values of measurement for the Quansys ELISA that were outside of the LOD range). 

### 3.5. Malaria Testing

The Quansys ELISA measures HRP2 as a biomarker measuring malarial infection. We compared HRP2 concentrations in 60 Burkina Faso children with completed qualitative testing of malaria parasitemia (Table 6). Considering the qualitative method as the reference, the specificity and sensitivity of the HRP2 biomarker measured on the Quansys ELISA to identify children with malaria was as follows: 72% sensitivity (true-positive rate) and 80% specificity (true-negative rate). The resulting Kappa coefficient is 0.680 (95% CI 0.488, 0.872), which is considered to indicate ‘good agreement’.

## 4. Discussion 

This study compares a newly established method (Quansys ELISA) with s-ELISA results from a single laboratory (VitMin lab) which has over the recent years been widely used in the field of surveys assessing vitamin A and iron deficiencies. As such, this study is not a method validation per se, but rather an evaluation to determine if results from a newly established method can be used interchangeably with those from an established lab. This is an important distinction to keep in mind, as for both the newly established method and the established approach, validation studies have been conducted [2,5]. Further, it is noteworthy that although the Quansys ELISA platform is on the market, the producer of the platform continues to further improve the analytical performance. As such, results presented here are of somewhat transient nature. 

Results from our comparison almost consistently indicate poor overall agreement of the two methods, both with regard to absolute biomarker concentrations and deficiency prevalence estimates. For most of the analytes, there is a relatively wide scattering between the two methods as can be seen from the Bland-Altmann plots. Moreover, as can be seen in Figure 1, Figure 2, Figure 3, Figure 4 and Figure 5 in the results section, there is also a systematic shift for several biomarkers, in particular for ferritin, sTfR, and CRP, and to some extent RBP. Such bias may be a result of different affinity of the antibodies in the two methods [14] and as such, if proving to be constant, could be justifiably adjusted, in particular if absolute true values could be established to adjust for. However, this shift is not consistent across the samples from the three countries such that from this work, no factor-adjustment to render the two methods more comparable can be proposed without reservation. Further, in comparison with a recently published report comparing the Quansys method to other methods based on samples from pregnant women in Niger [5], the shift varies again, despite some rough qualitative agreement (above or below the line of equality): the slopes (*y* = Quansys, *x* = comparing method) are 1.88*x* in that work vs. 2.08*x* for pooled Ferritin in our work, 1.70*x* vs. 2.48*x* for sTfR, 0.67*x* vs. 0.99*x* for CRP, and 0.47*x* vs. 0.63*x* for AGP; for RBP, this qualitative trend does not hold true: 0.84*x* vs. 1.12*x*. 

The observed lack of concordance may be attributed to the sample preparation. In 2017, the Quansys assay was validated using a Nigerian cohort of heparinized plasma samples [15]. The fact that serum and capillary samples were used in this study could account for some of the observed assay differences. Further studies would be warranted to further understand the impact of sample collection methods on both the s-ELISA and Quansys multiplex assay.

With regard to malaria parasitemia, 80% percent of the cases were truly positive and 72% were truly negative, when using the on-site rapid diagnostic test kit as the reference method. This results in a Kappa coefficient indicating good agreement. However, Kappa statistics are being used primarily for inter-observer comparability and as such, are tending to overestimate the comparability of biochemical methods. 

In terms of handling the Quansys ELISA, our impression is that the platform is relatively easy to use with minimal additional training for trained lab technicians. Instructions on assay preparation and completion are well-described in the assay handbook (available online and inside the kit). The kit contains the 96-well plate and all required reagents (calibrator, detection mix, substrate, sample diluents, and wash buffer). The Q-View™ software (Quansys Biosciences, Logan, UT, USA) is user-friendly and tutorials are available online to provide the user with additional guidance if needed. Despite the simple handling, there were some challenges faced and these revolve in particular around the platform’s automatic calculation of the LOD: this calibration is done for each new plate and a built-in algorithm fits a curve to the measured concentration of the calibrators. Particularly for ferritin, this led to difficulty in interpreting deficiency status on two plates, where the fitted curve resulted in estimations of ferritin concentrations below the threshold for defining iron deficiency in children (two plates produced *n* = 14/40 and *n* = 9/40 ferritin values defined as <15.16 μg/L and <14.13 μg/L, respectively; both below or just around the cut-off for iron deficiency of <12 μg/L and <15 µg/L, see Table 2). For these *n* = 23 samples, we could not confirm if children had a ferritin concentration below the 12 μg/L cut-off for deficiency. Through discussions with technical staff of Quansys, we identified options to ‘manually’ adjust the calibration curve and lower the LOD. However, we decided against presenting results of such manual adjustments, since this comparison was meant to be done for routine analyses and not a research-context. Instead, we present the result including and excluding samples where these challenges were faced. As previously mentioned, Quansys is working on improving the calibration curve and introducing quality control samples to reduce such issues.

As observed in several of the Bland-Altman plots, there appeared to be poorer agreement between values at the higher end (this was especially the case for ferritin (see Figure 1)). We recognize that most assays are developed with the goal to achieve high accuracy of measurement near the threshold that defines deficiency (for ferritin, ~12–15 μg/L). This is intended to optimize diagnostic accuracy with use of ferritin measurements. However, this is likely achieved with the consequence of decreased accuracy at higher values (e.g., ferritin > 150 μg/L). We considered whether or not we should exclude higher values of each analyte because of the poor agreement observed among values at the higher end. However, it was challenging to arbitrarily estimate what these higher values should be. This is also because the higher end threshold would likely vary from assay to assay, and would also depend on the controls used in each assay (which differ from lot to lot). Without a consensus on what higher end thresholds should be, and if we should exclude those values for the purposes of a method-comparison, we decided not to exclude any values. However, we raise this issue as one that requires further attention and solution, and note that the agreement between the methods in our study would likely have been higher if we excluded those higher (potentially inaccurate) values.

The strength of our comparison is that we had samples over a wide concentration range of most analytes available, samples from both women and children and from Asia and Africa. Although this is not an exhaustive representation, the samples certainly provide a certain heterogeneity in their origins. One possible limitation is that samples were analysed at different time points after obtaining them and during that time, they were stored under different conditions. This may have affected the analytes (concentration or chemical form) and thereby affected comparability. All analytes used in this work are proteins and are considered relatively stable for an extended period of time if kept frozen at −80 °C. For example, ferritin is very stable and serum can be frozen at −20 to −70 °C for several years without affecting sample quality [16]. Samples from Malaysia and Cambodia followed this standard, but this was not the case for Burkina Faso, where samples were stored at −20 °C for about 2 months and from then onwards at −40 °C. Further, the time between the two analyses was less than one year for samples from Malaysia and Burkina Faso, while it was two years for those from Cambodia, thus, we believe degradation of sample poses a very minor risk. Moreover, we suspect that degradation was not an issue as the Quansys concentrations tend to be higher than those obtained from the VitMin lab. The other important limitation is that this study is not using a method performance validation against a gold standard, but merely compares one newly-developed method with an established method. Thus, it would be inappropriate to say that one method is wrong and the other is right, both may have shifts from the true concentrations of the analytes. Yet, the VitMin Lab method has undergone several critical assessments and mostly yielded good scores [2,14]; plus, it is so widely used that it is almost setting a benchmark for new methods to be compared against. 

In our prior work, we measured serum ferritin concentrations using four different immunoassays, including Erhardt’s s-ELISA, in Cambodian women (*n* = 420) and Congolese children (*n* = 226) [14]. We observed differences in mean serum ferritin concentrations across assays, which were likely an inherent reflection of the different ferritin isoforms, antibodies, and calibrators used by these assays and labs [14]. Despite the differences in ferritin concentrations, iron deficiency prevalence was similar (and low) across the different ferritin methods [14]. Similarly, we suspect that some of the observed differences in ferritin concentrations are likely due to the different analytical (isoforms and antibodies) and calibration techniques between the two methods. 

In conclusion, we think it would be important to identify and publish the upper thresholds for both methods used here, so that users can easily understand where the methods’ limitations are in terms of accurate measurement, although we recognize this is difficult without the use of standard controls across all methods. With regard to the Quansys ELISA, although it has great potential to simplify laboratory analysis of ferritin, sTfR, RBP, CRP and AGP, there are still some weaknesses in the procedures that will need to be eliminated for routine use, in particular the automated calibration curves. In our use, it sometimes led to measured values below the established thresholds defining deficiency, in particular with ferritin. It would further be helpful if the test kits included certified quality control materials that would enable the user to spot inconsistencies relatively easily in routine use. An important caveat is that the systematic shift between the two methods is not constant for a given analyte in the samples from the three population in our study. More research is needed to establish such adjustment factors, if the comparability cannot be improved by changing the concentration of the capture antibodies. 

In its present form, the Quansys platform may be of interest to laboratories that have methods in place for analysing these analytes at small-scale and are looking for higher throughput options. In such a context, the laboratory may be able to cross-compare results on a regular basis and detect inconsistencies originating from the Quansys platform. For routine use in settings where such quality control options are not easily available, we conclude from our data that the risk of obtaining biased results is currently too high. 

## Figures and Tables

**Table 1:** Summary of the origin of the 180 serum samples included for method-comparison analysis.

Country	Population Group	Total *n* Available ^1^	Total *n* Analysed ^2^
Burkina Faso	Children aged 21–24 month	2100	60
Cambodia	Non-pregnant women aged 18–45 year	809	60
Malaysia	Non-pregnant women aged 18–45 year	200	60

^1^ Numbers are estimates, as some samples had insufficient volumes; ^2^ Purposively selected samples from all available samples in order to represent a range of values that were low, near the cut-off for deficiency, and high.

**Table 2:** Quality control data for the Quansys enzyme-linked immunosorbent assay (ELISA) for each of the five assays conducted (total *n* = 180).

	Analyte	ULOQ ^1^	LLOQ ^2^	LOD ^3^	Lowest Value (<)	Total *n* of Low Values	Highest Value (>)	Total *n* of High Values
Assay 1	Ferritin	110.16	1.39	0.067	<13.94	11/39	-	-
*n* = 40	STfR	118.49	0.17	0.065	-	-	-	-
	AGP	0.34	0.00059	0.00061	-	-	-	-
	CRP	17.87	0.029	0.0084	<0.29	3/40	-	-
	RBP	0.39	0.0016	0.00053	-	-	>3.9	2/40
	HRP2	1.03	0.0015	0.00026	<0.015	40/40	-	-
Assay 2	Ferritin	112.43	0.38	0.18	<3.84	3/40	-	-
*n* = 40	STfR	118.27	0.17	0.001	<1.70	2/40	-	-
	AGP	0.36	0.00053	0.00021	-	-	-	-
	CRP	6.97	0.029	0.0065	<0.29	2/40	-	-
	RBP	0.39	0.0015	0.00027	-	-	>3.86	2/40
	HRP2	1.03	0.004	0.00092	<0.040	40/40	-	-
Assay 3	Ferritin	111.94	1.52	0.081	<15.16	14/40	-	-
*n* = 40	STfR	116.62	0.16	0.84	-	-	-	-
	AGP	0.37	0.00059	0.0004	-	-	-	-
	CRP	19.86	0.03	0.001	-	-	-	-
	RBP	1.06	0.0043	0.001	-	-	-	-
	HRP2	1.05	0.0014	0.0056	<0.014	29/40	>10.45	8/40
Assay 4	Ferritin	112.31	1.43	0.065	<14.31	9/20	-	-
*n* = 20	STfR	115.36	0.16	0.026	-	-	-	-
	AGP	0.35	0.0015	0.00056	-	-	-	-
	CRP	18.45	0.26	0.0018	<0.26	-	-	-
	RBP	0.11	0.0016	0.0016	-	-	>1.08	18/20
	HRP2	1.02	0.0015	0.00065	<0.015	16/20	-	-
Assay 5	Ferritin	113.16	0.4	0.24	<3.97	5/40	-	-
*n* = 40	STfR	117.21	1.36	0.008	-	-	-	-
	AGP	0.36	0.0014	0.00081	-	-	-	-
	CRP	18.74	0.027	0.01	-	-	-	-
	RBP	0.38	0.0042	0.002	-	-	-	-
	HRP2	1.05	0.0045	0.000034	<0.045	26/40	>10.51	6/40

^1^ ULOQ: the highest standard curve point that can still be used for quantification; the highest concentration of an analyte that can be accurately measured; ^2^ LLOQ: the lowest point standard curve point that can still be used for quantification; the lowest concentration of an analyte that can be accurately measured; ^3^ LOD: limit of detection, the lowest concentration level that can be determined to be statistically different from a blank at a 99% confidence level.

**Table 3:** Nutritional biomarkers and deficiency prevalence of the women and children based on s-ELISA and Quansys measurements both within and outside of the limit of detection (LOD) range ^1^.

	Burkina Faso Children			Cambodian Women			Malaysian Women		
	s-ELISA *n* = 60	Quansys_EXCL ^2^ *n* = 47–60	Quansys_INCL ^3^ *n* = 60	s-ELISA *n* = 60	Quansys_EXCL ^2^ *n* = 39–60	Quansys_INCL ^3^ *n* = 59–60	s-ELISA *n* = 60	Quansys_EXCL ^2^ *n* = 51–60	Quansys_INCL ^3^ *n* = 60
Ferritin, μg/L	20.5 (8.9, 44.8)	34.6 (16.9, 91.0)	22.5 (14.5, 62.6)	29.3 (16.9, 59.8)	54.9 (31.0, 105.7)	31.0 (14.3, 73.5)	47.1 (23.2, 81.9)	62.6 (29.7, 118.6)	54.6 (17.8, 96.0)
<12 or <15 μg/L ^4^, *n* (%)	19/60 (32%)	8/47 (17%)	13/60 (22%)	14/60 (23%)	0/39 (0%)	0/59 (0%)	11/60 (18%)	3/51 (6%)	6/60 (10%)
STfR, mg/L	10.4 (8.3, 15.8)	30.7 (22.8, 49.5)	30.7 (22.8, 49.5)	6.6 (4.8, 9.6)	8.5 (6.6, 14.6)	8.5 (6.6, 14.6)	5.2 (4.2, 6.0)	4.7 (3.6, 5.8)	4.7 (3.6, 5.8)
>8.3 mg/L, *n* (%)	45/60 (75%)	60/60 (100%)	60/60 (100%)	21/60 (35%)	30/60 (50%)	30/60 (50%)	5/60 (8%)	10/58 (17%)	10/60 (10%)
AGP, g/L	1.25 (0.86, 1.82)	1.25 (0.97, 1.56)	1.25 (0.97, 1.56)	0.57 (0.44, 0.76)	0.58 (0.50, 0.69)	0.58 (0.50, 0.69)	0.57 (0.49, 0.72)	0.81 (0.65, 0.99)	0.81 (0.65, 0.99)
>1 g/L, *n* (%)	39/60 (65%)	40/60 (67%)	40/60 (67%)	9/60 (15%)	6/60 (10%)	6/60 (10%)	2/60 (3%)	15/60 (25%)	15/60 (25%)
CRP, mg/L	3.2 (0.9, 9.2)	7.3 (2.0, 16.3)	7.3 (2.0, 16.3)	0.4 (0.2, 0.8)	1.4 (0.9, 3.0)	1.4 (0.7, 2.8)	0.78 (0.35, 2.16)	3.0 (1.3, 8.5)	2.7 (0.9, 8.3)
>5 g/L, *n* (%)	25/60 (42%)	35/60 (58%)	35/60 (58%)	4/60 (7%)	10/57 (18%)	10/60 (17%)	6/60 (10%)	19/56 (34%)	19/60 (32%)
RBP, μmol/L, mean ± SD	0.83 ± 0.29	0.88 ± 0.33	0.88± 0.33	1.62 ± 0.62	1.51 ± 0.59	1.59 ± 0.72	1.44 ± 0.32	1.97 ± 0.63	2.03 ± 0.71
<0.7, μmol/L, *n* (%)	12/60 (20%)	23/60 (38%)	23/60 (38%)	1/60 (2%)	1/58 (2%)	1/60 (2%)	0/60 (0%)	0/58 (0%)	0/60 (0%)

^1^ Values are median (interquartile range; IQR) unless otherwise indicated. AGP, α^1^-acid-glycoprotein; CRP, C-reactive protein; LOD, limit of detection; RBP, retinol-binding protein; SD, standard deviation; sTfR, soluble transferrin receptor; ^2^ Excludes values of measurement for the Quansys ELISA that were outside of the LOD range; ^3^ Includes all values of measurement for the Quansys ELISA, however, for values outside of the LOD range, values were included in the analysis at the value of the lowest or highest value of the LOD (e.g., for a ferritin value measured as <1.5 μg/L on the Quansys ELISA, we included in the analysis as a value of 1.5 μg/L); ^4^ Ferritin <12 μg/L for children and <15 μg/L for women.

**Table 4:** Linear trend equations for each population group for the comparison between each of the two methods.

	Burkina Faso	Cambodia	Malaysia	Pooled ^1^
Ferritin_EXCL ^2^	1.58*x* − 4.66	2.26*x* − 57.54	2.84*x* − 79.32	2.25*x* − 42.79
Ferritin_INCL ^3^	1.55*x* − 2.78	2.00*x* − 30.93	2.56*x* − 54.31	2.08*x* − 27.59
STfR ^2^	1.75*x* + 17.19	2.07*x* + 4.55	1.62*x* − 3.57	2.48*x* − 3.04
RBP ^2^	0.97*x* + 0.07	0.96*x* − 0.02	1.55*x* − 0.25	1.12*x* + 0.04
CRP ^2^	1.05*x* + 3.76	0.66*x* + 2.13	1.47*x* + 2.22	0.99*x* + 2.88
AGP ^2^	0.54*x* + 0.56	0.56*x* + 0.26	0.86*x* + 0.30	0.64*x* + 0.36

^1^ Pooled results are trend estimates for Burkina Faso, Cambodia, and Malaysia combined; ^2^ Excludes values of measurement for the Quansys ELISA that were outside of the LOD range; ^3^ Includes all values of measurement for the Quansys ELISA (within/outside of the LOD range).

**Table 5:** Bias, limits of agreement, and correlation coefficients of ferritin, soluble transferrin receptor (sTfR), α^1^-acid glycoprotein (AGP), C-reactive protein (CRP), and retinol-binding protein (RBP) concentrations comparing the s-ELISA and the Quansys ELISA kit.

Participants	All Samples ^1^					Only Samples Within LOD Range ^2^				
	Total, All	Bias	Limits of Agreement	Pearson’s Coefficient	Concordance (95% CI)	Within Range ^1^	Bias	Limits of Agreement	Pearson’s Coefficient	Concordance (95% CI)
	*n*	mean ± SD	±1.96 SD	*r*	*ρ*_c_ (±1.96 SD)	*n*	mean ± SD	±1.96 SD	*r*	*ρ*_c_ (±1.96 SD)
Ferritin, μg/L										
Burkina Faso children	60	14.7 ± 36.0	−55.8, 85.3	0.81	0.62 (0.51, 0.71)	47	17.8 ± 40.1	−60.7, 96.3	0.78	0.57 (0.43, 0.68)
Cambodian women ^3^	59	18.3 ± 84.4	−147.1, 183.7	0.81	0.55 (0.45, 0.63)	39	27.1 ± 103.0	−174.8, 229.0	0.80	0.48 (0.36, 0.59)
Malaysian women	60	29.3 ± 104.6	−175.8, 234.3	0.76	0.40 (0.31, 0.48)	51	34.1 ± 112.9	−187.3, 255.4	0.76	0.36 (0.27, 0.45)
STfR, mg/L										
Burkina Faso children	60	26.5 ± 21.8	−16.1, 69.1	0.42	0.09 (0.03, 0.14)	60	26.5 ± 21.8	−16.1, 69.1	0.42	0.09 (0.03, 0.14)
Cambodian women	60	5.2 ± 10.4	−15.2, 25.6	0.91	0.62 (0.54, 0.69)	60	5.2 ± 10.4	−15.2, 25.6	0.91	0.62 (0.54, 0.69)
Malaysian women	60	0.1 ± 3.3	−6.3, 6.5	0.92	0.78 (0.72, 0.83)	58	0.2 ± 3.3	−6.3, 6.6	0.91	0.78 (0.72, 0.83)
AGP, g/L										
Burkina Faso children	60	−0.1 ± 0.4	−0.9, 0.8	0.67	0.65 (0.49, 0.77)	60	−0.1 ± 0.4	−0.9, 0.8	0.67	0.65 (0.49, 0.77)
Cambodian women	60	−0.04 ± 0.2	−0.5, 0.4	0.94	0.82 (0.77, 0.86)	60	−0.04 ± 0.2	−0.5, 0.4	0.94	0.82 (0.77, 0.86)
Malaysian women	60	0.2 ± 0.2	−0.1, 0.6	0.66	0.41 (0.27, 0.54)	60	0.2 ± 0.2	−0.1, 0.6	0.66	0.41 (0.27, 0.54)
CRP, mg/L										
Burkina Faso children	60	4.1 ± 5.5	−6.7, 14.8	0.83	0.73 (0.61, 0.82)	60	4.1 ± 5.5	−6.7, 14.8	0.83	0.73 (0.61, 0.82)
Cambodian women	60	1.4 ± 4.0	−6.5, 9.3	0.83	0.79 (0.68, 0.86)	57	1.5 ± 4.1	−6.6, 9.6	0.83	0.79 (0.68, 0.86)
Malaysian women	60	3.1 ± 2.9	−2.6, 8.7	0.95	0.74 (0.65, 0.81)	56	3.3 ± 2.9	−2.4, 8.9	0.95	0.73 (0.64, 0.80)
RBP, μmol/L										
Burkina Faso children	60	0.04 ± 0.2	−0.3, 0.4	0.85	0.83 (0.74, 0.90)	60	0.04 ± 0.2	−0.3, 0.4	0.85	0.83 (0.74, 0.90)
Cambodian women	60	−0.04 ± 0.4	−0.8, 0.8	0.83	0.82 (0.72, 0.89)	58	−0.05 ± 0.4	−0.8, 0.7	0.74	0.72 (0.58, 0.82)
Malaysian women	60	0.6 ± 0.5	−0.3, 1.6	0.79	0.38 (0.27, 0.48)	58	0.5 ± 0.4	−0.3, 1.4	0.79	0.40 (0.28, 0.50)

^1^ Includes all values of measurement for the Quansys ELISA, however, values outside of the LOD range were included in the analysis at the value of the lowest or highest value of the LOD (e.g., for ferritin measured as <1.5 μg/L on the Quansys ELISA, we included in the analysis as a value of 1.5 μg/L). AGP, α^1^-acid-glycoprotein; CI, confidence interval; CRP, C-reactive protein; LOD, limit of quantification; RBP, retinol-binding protein; SD, standard deviation; sTfR, soluble transferrin receptor; ^2^ Excludes values of measurement for the Quansys ELISA that were outside of the LOD range; ^3^ *n* = 1 sample failed the ferritin Quansys ELISA assay (was reported as incalculable). 3.5. Bland-Altman plots comparing the s-ELISA and the Quansys ELISA kit for samples within the LOD range (excludes values for the Quansys ELISA that were outside of the LOD range).

**Table 6:** Accuracy of the HRP2 biomarker measured on the Quansys ELISA to identify Burkina Faso children with malaria.

	Malaria Diagnosis Confirmed by the Rapid Diagnostic Test (Reference)			
Quansys HRP2		Yes—Malaria	No—Malaria	Total
	Positive (HRP2 > 1)	True-positive 18/25 (72%)	False-positive 7/35 (20%)	25
	Negative (HRP2 < 1)	False-negative 7/25 (28%)	True-negative 28/35 (80%)	35
	Total	25	35	
